# Letter in response to case “Variants in the SARS2 gene cause HUPRA syndrome with atypical features: two case reports and review of the literature

**DOI:** 10.1093/omcr/omae153

**Published:** 2024-12-10

**Authors:** Ruth Belostotsky

**Affiliations:** Shaare Zedek Medical Center, Pediatric Nephrology Department, Shmuel Beyth St 12, Jerusalem, 9103102, Israel

Dear editor

The article by Lahham E.E., Hassasneh J.J., Adavi D.O., Ismail M.K. ‘Variants in the SARS2 gene cause HUPRA syndrome with atypical features: two case reports and review of the literature.’ [Oxf Med reports. November 28, 2023;2023(11)] describes two Palestinian girls from the same village with two different pathogenic homozygous variants of the SARS2 gene.

About 13 years ago we diagnosed a new multisystemic mitochondrial cytopathy (HUPRA syndrome) in two infants from a consanguineous Palestinian kindred living in a single village [1]. We confirmed that HUPRA syndrome was due to bi-allelic mutations in the SARS2 gene. Since then, the Israeli Ministry of Health introduced screening of carriers of the c.1169A>G (p.Asp390Gly) mutation for genetic counseling in couples at risk of the HUPRA syndrome, in a specific village near Jerusalem, Israel. In the ensuing years, we diagnosed a few more patients with the same mutation from the same village. Thus, we were very much surprised with the observation of the authors: ‘Two of our patients were found to have pathogenic homozygous variants of the SARS2 gene (c.1175A>G(p.Asp392Gly)) and (c.1169A>G(p.Asp390Gly)), in exon13.’ It seems strange that two girls from the same village have different homozygous mutations, given the extreme rarity of this disease. Moreover, in transcript ENST00000221431.6 SARS2-001 there is a C rather than an A in position c.1175, and it encodes Pro instead of Asp at position p.392.

Since I did not receive explanations from the authors, I undertook my own search and found out that the same genomic triplet [g.33549-51] encodes p.Asp390 in transcript ENST00000221431.6 SARS2-001 in exon 13 and p.392 in exon 14 in alternative transcript ENST00000600042.1 SARS2-003.

Thus, the authors erroneously interpreted the same mutation in two different cases and in fact published data from an already described cohort of patients from a consanguineous kindred living in an Israeli village.

Sincerely,

Ruth Belostotsky, Shaare Zedek Medical Center, Jerusalem, Israel.

## Conflicts of interest

The author does not have conflicts of interest.
